# Functional Compromise Cohort Study (FCCS): Sarcopenia is a Strong Predictor of Mortality in the Intensive Care Unit

**DOI:** 10.1007/s00268-017-4386-8

**Published:** 2017-12-29

**Authors:** P. A. de Hoogt, K. W. Reisinger, J. J. W. Tegels, J. W. A. M. Bosmans, F. Tijssen, J. H. M. B. Stoot

**Affiliations:** 1 Department of Surgery, Maastricht University Medical Center, P. Debyelaan 25, 6202 AZ Maastricht, The Netherlands; 2 Department of Surgery, Zuyderland Medical Center, Sittard-Geleen, The Netherlands; 3 Department of Anesthesiology, Maastricht University Medical Center, Maastricht, The Netherlands

## Abstract

**Background:**

Functional compromise in elderly patients is considered to be a significant contributing factor in increased postoperative morbidity and mortality. It is described as a state of reduced physiologic reserves including, e.g., sarcopenia, cachexia, malnutrition and frailty with increased susceptibility to adverse health outcomes. Aim of this study was to investigate the association of sarcopenia with mortality in ICU patients.

**Methods:**

A retrospective analysis of a total of 687 patients admitted to the ICU from January 2013 until December 2014 was performed. Indirect measurements of functional compromise in these patients were conducted. Sarcopenia was assessed using the L3 muscle index by using Osirix© on computed tomography scans. Groningen Frailty Indicator (GFI) and Short Nutritional Assessment Questionnaire (SNAQ) scores were extracted from the digital patient filing system and were used to assess frailty and nutritional status. These factors were analyzed using logistic regression analysis as predictor for in-hospital mortality and 6-month mortality, which was the primary endpoint along with other secondary outcome measures.

**Results:**

Age was an independent predictor of in-hospital mortality, OR 1.043 (95% CI 1.030–1.057, *p* < 0.001). Analysis of sarcopenia showed OR 2.361 (95% CI 1.138–4.895, *p* = 0.021), for GFI OR 1.012 (95% CI 0.919–1.113, *p* = 0.811) and for SNAQ OR 1.262 (95% CI 1.091–1.460, *p* = 0.002).

**Conclusion:**

This study shows a promising role for the sarcopenia score as a predictor of mortality on the ICU, based upon CT imaging at L3 level and SNAQ score. Further research is necessary to test this in larger cohorts and to develop a possible instrument to predict mortality in the intensive care unit.

## Introduction

Admission to the intensive care unit (ICU) is associated with high mortality rates up to 19% [1–3]. Recent studies show a mortality rate of 11.3% in 2010–2012 in the USA [4, 5]. Moreover, ICU admissions lead to impaired quality of life and morbidity such as cachexia, critical illness neuropathy, aspiration pneumonia, ventilator pneumonia and pseudomonas infections [6–10]. Also, ICU treatment is costly, and prolonged treatment for elderly or frail patients may not always be in the best interest of this group of patients. Therefore, tools are needed to predict outcome in ICU patients. In response to this need, several scoring systems have been developed to predict mortality in the ICU. Although most of them are used as benchmarking tools, none of them have been proven unequivocally effective for this purpose [9–12]. Frailty is defined as a state of reduced physiologic reserves associated with increased susceptibility to adverse health outcomes [13, 14]. Important elements are weight loss, low muscle strength, reduced physical activity, decreased walking speed and malnutrition. Recently, frailty had been shown to be a contributing factor in worse outcomes after gastric and colorectal surgery as well as for surgical ICU patients [14–16]. Furthermore, frailty has been associated with a lower survival and higher health resource utilization in patients admitted to the intensive care unit measured by markers of frailty such as the frailty phenotype (FP) and the clinical frailty score (CFS) [7, 17]. Elderly patients are likely to have more comorbidity and may receive overtreatment with a minimal chance of recovery. Several tools have been reported to determine the elements of frailty, like the Groningen frailty score (GFI), Clinical Frailty Scale (CFS), Fried’s frailty score, frailty phenotype, Tilburg Frailty Indicator (TFI), Edmonton Frail Scale and L3 muscle index [16, 18–22].

When reviewing any patient, but specifically elderly patients, functional compromise is to be evaluated. Functional compromise is defined as a condition, which includes frailty, but also other components such as sarcopenia, cachexia, malnutrition, vulnerability and fatigue [13, 14, 17, 21]. As a reflection of muscle depletion and malnutrition, sarcopenia has been shown to be a predictive for adverse outcome after surgery [14, 16]. Sarcopenia is defined as an involuntary loss of skeletal muscle mass. This can occur in normal weight, overweight and obese patients and therefore is not equal to weight loss or cachexia [8, 23–25]. Muscle mass can be easily measured on an abdominal CT imaging, and sarcopenia can then be assessed by the L3 index [8, 23–26].

Other parts of functional compromise such as nutritional status and overall functioning can be reviewed by different questionnaires like the MUST or SNAQ score [18, 19].

The aim of our study was to investigate whether sarcopenia alone or as part of frailty is associated with mortality in patients admitted to the intensive care unit.

## Methods

### Legal and ethical committee

Permission for this research was obtained from the local ethics and legal committee of the Zuyderland Medical Centre and have been performed in accordance to the Declaration of Helsinki and its later amendments.

### Study population

**Fig. 1:**
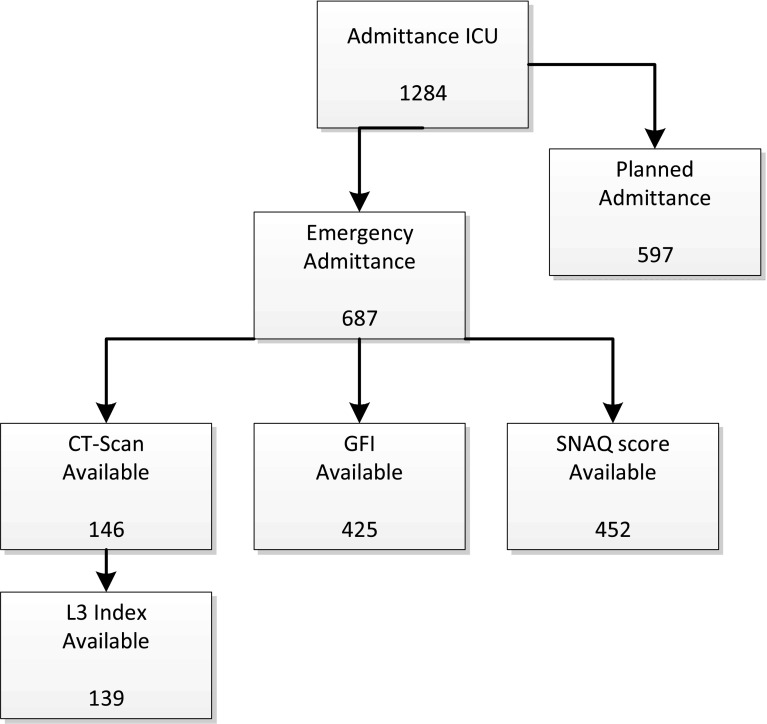
Inclusion of study population. *ICU* intensive care unit of Zuyderland MC Sittard-Geleen in 2013 and 2014, *GFI* Groningen Frailty Indicator, *SNAQ* Short Nutritional Assessment Questionnaire, *L3 index* L3 muscle index calculated by measuring muscle surface at lumbar vertebra 3 corrected for BMI

All consecutive patients who were admitted because of an emergency to the intensive care unit of the Zuyderland Medical Centre, location Sittard-Geleen, from January 2013 until December 2014 were included. Exclusion criteria were: patients who were admitted to the ICU as part of elective surgery and cases in which predictor variables of interest (GFI score, SNAQ score and abdominal CT scan) were not complete. CT scans performed within 3 months prior to or after admission were used to calculate L3 muscle index. A flowchart of the study population is provided in Fig. 1.

### Outcome measures

The primary outcome in this study is mortality (both in-hospital and 6-month mortality). Mortality was investigated by assessing the electronic patient files and cross-checked by consulting the municipal administration database.

### Frailty and nutritional status

At primary admission, a patient interview took place in the ward as part of standard procedure. In this interview, standardized questionnaires regarding current health and nutritional status were administered, including the Groningen Frailty Indicator (GFI) and Short Nutritional Assessment Questionnaire (SNAQ). The GFI score covers seven domains of frailty: mobility, cognition, vision and hearing impairment, nutritional status, comorbidities, social and psychological condition (see Appendix 1 for the full questionnaire). For each question the maximum of one point can be scored.

Malnutrition was assessed by the SNAQ score [16, 18]. In case of an aberrant SNAQ score (2), intervention by a dietitian was followed/arranged (see Appendix 2 for the full questionnaire).

### Sarcopenia

Sarcopenia was determined by measuring the L3 index on an abdominal computed tomography (CT) scan performed between 3 months before admission and 3 months after discharge. Total cross-sectional surface area (cm2) measurements were performed at the level of the third lumbar vertebra (L3) on two consecutive transversal slides on which both transverse processes were visible [25, 27]. Measurements were performed using Osirix© open-source software (version 6.0.1 32-bit version) in a semi-automated fashion. By setting tissue of interest, thresholds were set at −29 to 150 HU for skeletal muscle total cross-sectional area of adipose tissue, and skeletal muscle was measured. Hand adjustment of the selected areas was performed if necessary, and the muscle area was calculated automatically, as previously described [14, 24, 25, 28].

### Severity of disease

The APACHE score was originally designed as a quantification method for the severity of disease in patients admitted to the ICU. This score is calculated by entering a number of different parameters, PaO2, temperature, mean arterial pressure, arterial pH, heart rate, respiratory rate, Glasgow Coma Scale and blood analysis for sodium, potassium, creatinine, hematocrit and white blood cell count. These measurements should be conducted within the first 24 h of admission on the ICU, and the score cannot be changed after this period. The latest version, APACHE IV, has been improved by using a new logistical regression equation, different set of variables and statistical modeling, which increased the accuracy of this instrument [5, 11, 12] and was obtained from the NICE registration for the purpose of this study.

### In-hospital mortality predictor

The simplified acute physiologic score (SAPS) can be used to predict hospital mortality. Parameters to calculate the SAPS score are: age, heart rate, systolic blood pressure, temperature, Glasgow Coma Scale (GCS), mechanical ventilation or CPAP, PaO2, FiO2, urine output, blood urea nitrogen, sodium, potassium, bicarbonate, bilirubin, white blood cell count, chronic disease and type of admission. Patients with higher SAPS scores have a higher mortality risk [10]. The current version of the SAPS score instrument is SAPS II; however, recently SAPS III was validated [9]. Recent studies show a good discrimination by SAPS III [29]. At the time of the study, SAPS II was used in the ICU, and this score is therefore taken into account during analysis.

### Acquiring data

Data were acquired using fully electronic patient information system(SAP) where data were entered prospectively during admission. For this study, data were obtained retrospectively. By using queries, conducted by the IT department, data from patients from January 2013 until December 2014 who were admitted to the ICU were extracted from the system. If specific data were unavailable, an additional manual search was conducted into individual patient records. Moreover, specific ICU data were extracted from the NICE database, which consists of necessary parameters to construct APACHE and SAPS scores.

### Statistical analysis

Frequencies are presented as absolute numbers and percentages. Continuous data are presented as mean (standard error of the mean). Statistical analyses were performed using IBM SPSS Statistics, Version 23, Armonk, NY, USA. Baseline characteristics were evaluated by using the Levene’s test as part of the independent sample *T* test to assess the equality of variance in our study group. Odds ratios (ORs) and 95% confidence intervals (CIs) were calculated by a logistic regression analysis. The level of statistical significance was set at *p* < 0.05. For the calculation of significant differences between groups of mortality, univariate analyses with clinically relevant parameters were performed. Significant predictors (*p* < 0.05) or predictors showing a trend toward significance (0.05 ≤ *p* < 0.20) based on univariate analysis were entered into multivariate logistic regression analysis.

## Results

### Baseline characteristics

Baseline characteristics (gender, length, weight, age and BMI) were analyzed and showed a difference in the factor age, as shown in Table 1. Since age was a significant predictor of mortality (*p* < 0.001), age at time of admission was entered as variable in the univariate analysis.

**Table 1:** Patient characteristics

	Number of patients (%)
Sex	
Male	367 (53.4)
Female	320 (46.6)
Age (years)	
>70	296 (43.1)
BMI (kg/m^2^)	
>30	50 (20.7)
25–30	84 (34.9)
20–25	88 (36.5)
15–20	15 (6.2)
<15	4 (1.7)
ASA	
I	24 (14.9)
II	85 (52.8)
III	46 (28.6)
IV	6 (3.7)
CAT scans	
Number of CAT scans	146
Mean	4.36 days after admission
Min/max	−176, 130
Admission diagnosis	
Pulmonary	113 16.7 (%)
Cardiac	111 16.4 (%)
Metabolic	97 14.3 (%)
Neurologic	22 3.2 (%)
Vascular	68 10.0 (%)
Hemorrhagic	41 6.1 (%)
Gastrointestinal	96 14.2 (%)
Head trauma	4 0.6 (%)
Sepsis	46 6.8 (%)
Other	79 11.7 (%)
Total	677

### Mortality

Of 687 patients in the emergency admission group, 207 patients (19%) died in the period of admission (Table 2). In the univariate analyses, SAPS II (*p* < 0.001), APACHE IV (*p* < 0.001), age at admission (*p* < 0.001), sarcopenia (*p* = 0.021) and SNAQ (*p* = 0.002) were significant predictors of mortality in the intensive care unit. Multivariate logistic regression of these predictors revealed all of these were not statistically significant.

**Table 2:** Logistic regression analysis for risk factors of mortality

	Mortality rate	Univariate analysis		Multivariate analysis	
		Odds ratio	*p*	Odds ratio	*p*
(a)					
Sarcopenia					
No	14/80 (18%)	1		1	
Yes	20/59 (34%)	2.418 (1.098–5.324)	0.028	2.014 (0.153–26.553)	0.595
GFI	83/425 (20%)	0.941 (0.837–1.058)	0.307		
GFI ≥ 3					
No	62/314 (20%)	1			
Yes	21/111 (19%)	0.948 (0.547–1.644)	0.850		
GFI ≥ 4					
No	69/348 (20%)	1			
Yes	14/77 (18%)	0.899 (0.476–1.698)	0.742		
SNAQ	76/452 (17%)	1.212 (1.028–1.429)	0.022	2.669 (0.689–10.342)	0.155
SNAQ ≥ 3					
No	58/386 (15%)	1			
Yes	18/66 (27%)	2.121 (1.153–3.901)	0.016	0.111 (0.000–37.119)	0.459
Gender					
Female	60/320 (19%)	1			
Male	70/367 (19%)	0.979 (0.668–1.436	0.979		
Age	130/687 (19%)	1.039 (1.024–1.054)	<**0.001**	1.027 (0.863–1.223)	0.761
Age > 70					
No	52/391 (13%)	1			
Yes	78/296 (26%)	2.333 (1.579–3.445)	<**0.001**	2.007 (0.035–113.520)	0.735
ASA					
I	4/24 (17%)	1.653 (0.668–4.093)	0.277		
II	7/85 (8%)	0.746 (0.396–1.405)	0.364		
III	3/46 (7%)	0.816 (0.335–1.988)	0.655		
IV	2/6 (33%)	16.868 (4.056–70.124)	<**0.001**	3.030 (0.079–115.755)	0.551
BMI > 25 kg/m^2^					
No	17/107 (16%)	1			
Yes	27/134 (20%)	1.336 (0.685–2.607)	0.396		
APACHE IV	107/608 (18%)	1.043 (1.034–1.052)	<**0.001**	0.980 (0.876–1.096)	0.721
SAPS II	107/608 (18%)	1.070 (1.055–1.085)	<**0.001**	1.003 (0.833–1.208)	0.975
LODS	107/608 (18%)	1.358 (1.272–1.449)	<**0.001**	1.720 (0.625–4.729)	0.294
(b)					
Sarcopenia					
No	19/80 (24%)	1		1	
Yes	25/59 (42%)	2.361 (1.138–4.895)	0.021	3.005 (0.321–28.145)	0.815
GFI	117/425 (28%)	1.012 (0.919–1.113)	0.811		
GFI ≥ 3					
No	85/314	1			
Yes	32/111	1.091 (0.675–1.764)	0.721		
GFI ≥ 4					
No	95/348 (27%)	1			
Yes	22/77 (29%)	1.065 (0.616–1.842)	0.821		
SNAQ	118/452 (26%)	1.262 (1.091–1.460)	0.002	2.256 (0.620–8.204)	0.217
SNAQ ≥ 3					
No	90/386 (23%)	1			
Yes	28/66 (42%)	2.423 (1.409–4.168)	0.001	0.012 (0.000–64.949)	0.314
Gender					
Male	106/367 (29%)	1			
Female	79/320 (25%)	0.807 (0.575–1.132)	0.217		
Age	185/687 (27%)	1.043 (1.030–1.057)	0.000	0.888 (0.767–1.029)	0.114
Age > 70					
No	75/391 (19%)	1			
Yes	110/296 (37%)	2.492 (1.765–3.518)	0.000	13.621 (0.420–441.971)	0.141
ASA					
I	5/24 (21%)	1.478 (0.507–4.307)	0.474		
II	8/85 (9%)	0.398 (0.168–0.940)	0.036	0.316 (0.044–2.287)	0.254
III	8/46 (17%)	1.162 (0.481–2.809)	0.738		
IV	3/6 (50%)	5.786 (1.111–30.120)	0.037	0.685 (0.019–25.163)	0.837
BMI > 25 kg/m^2^					
No	27/107 (25%)	1			
Yes	31/134 (23%)	0.892 (0.493–1.613)	0.705		
APACHE IV	154/608 (25%)	1.042 (1.033–1.050)	<**0.001**	0.991 (0.881–1.115)	0.882
SAPS II	154/608 (25%)	1.068 (1.054–1.081)	<**0.001**	1.022 (0.855–1.222)	0.810
LODS	154/608 (25%)	1.353 (1.272–1.438)	<**0.001**	1.359 (0.522–3.540)	0.530

Bold values indicate statistical significance

Definition of mortality: in-hospital mortality (a) and/or 6-month mortality (b)

Values in parentheses are 95% confidence intervals

### Frailty and nutrition

When reviewing SNAQ scores in perspective of in-hospital mortality, a SNAQ score of at least three points resulted in an OR of 2.121 (95% CI 1.153–3.901, *p* = 0.016). To illustrate this, 27% of the higher scoring group died versus 15% in the group scoring lower than three.

GFI did not show a clear association between higher scores and mortality. Both scores of ≥3 and ≥4 were tested and were not significant: 20 versus 19%, OR of 0.948 (95% CI 0.0547–1.644, *p* = 0.850) and 20 versus 18%, OR 0.899 (95% CI 0.476–1.698, *p* = 0.742), respectively. This was the case for both in-hospital mortality and 6-month mortality.

### Sarcopenia

Of 687 patients included in this study, 146 CAT scans were available for measurements at the L3 level. In this group, seven patients had unknown BMI; therefore, L3 index could be calculated for 139 patients. When reviewing in-hospital mortality, 34% of the patients marked as sarcopenic by the use of BMI corrected L3 muscle index died, while in the other group this was 18%, univariate analysis (*p* = 0.028). When considering 6-month mortality, this difference is 42% in the sarcopenic group versus 24% in the non-sarcopenic group (*p* = 0.021).

## Discussion

This study showed significant differences in mortality for patients with a higher SNAQ score and sarcopenia (L3 index measure). GFI showed no significance in predicting mortality. Therefore, this study shows sarcopenia and nutritional status by evaluation of SNAQ score are important factors in predicting mortality in patients admitted to the ICU.

In 2013, 82.161 patients were admitted in the ICU in the Netherlands, of whom 10.697 (13%) died. Fifty-seven percent (6.052/10.697) of the deceased patients were older than the age of 70. In this group of elderly patients, mortality rate was 18.5% compared to 9.4% in the patients younger than 70 years old [30]. Although this difference can be partially explained by age, when corrected for this factor, the difference in mortality remains, indicating that other factors might contribute to the higher risk. Geriatric frailty might better predict the increased risk of adverse outcomes in elderly patients than age alone. The preferred method of determining frailty is by a comprehensive geriatric assessment. However, this is very time- and resource-consuming. Therefore, several questionnaires were developed like Edmonton Frail Scale, Fried’s frailty, frailty phenotype, clinical frailty score, Tilburg Frailty Indicator and Groningen Frailty Indicator.

Previous studies showed GFI to be a significant predictor for complications and mortality in colorectal and gastric cancer patients [16, 25]. In this study, patients were considered to be frail when scoring three or higher on the GFI scoring list. This is according to the methods of previous studies [16, 25].

Based upon previous findings, we expected to see a significant difference in our population. Reason for this difference might be an incomplete filing of the GFI score causing a lower reported score. It can be hypothesized that if the GFI scores were completely conducted and not 0 was reported for missing data, it would be possible to better discriminate between patients who are considered to be frail and those that are not. Introduction of the use of GFI was done just before our study start point, which may explain the findings since familiarity with the GFI score might not have been optimal, resulting in underestimation of frailty.

More expertise in our center can be found in using the SNAQ questionnaire that was used to evaluate nutritional deficiencies. This study showed significant prediction of mortality in ICU patients using the SNAQ which is in accordance with the current literature [16, 18, 25, 28].

Both SNAQ and GFI questionnaires are easy to obtain and can therefore be easily implemented into standard clinical practice. Since many studies, including the study reported here, have shown SNAQ as a evident predictor of mortality, we would like to advocate to use this questionnaire in every patient, not when suspected to be frail or admitted to the ICU.

Although univariate analysis shows significant predictors like SNAQ and sarcopenia, multivariate analysis shows no significance. Possible explanation for this result is confounding of age or the incomplete database.

This study is the first study to compare frailty scores in the form of L3 muscle index, GFI and SNAQ with current benchmarking and mortality predicting scores as APACHE III, APACHE IV and SAPS II. However, the main limitations of this study are its single-center design and retrospective analysis. As a consequence of this design, the dataset was partly incomplete (GFI score). One might hypothesize that with a complete dataset, the outcome, the association between frailty and mortality, was even stronger.

Apart from the incomplete filing of the GFI and SNAQ in the critically ill patients, not all patients had an available CT scan for evaluation of muscle mass at L3 level.

Therefore, taking into account the retrospective study at a single center and a medium sized intensive care unit, the population was limited by the severity of ill patients.

Possibly a multicentered, prospective study could be useful to investigate the hypothesis in a more diverse population with a completely filled GFI and SNAQ scores.

## Conclusion

This retrospective, single-center study shows a promising role for the “Zuyderland Sarcopenia Score,” which consists of L3 sarcopenia measurement and the SNAQ score. Further research is necessary to determine further sensitivity and specificity and design a possible instrument to predict mortality in the intensive care unit.

## Appendix 1: Items of Groningen Frailty Indicator Score

### Mobility

Is the patient able to carry out these tasks single-handed without any help? (The use of help resources, such as walking stick, walking frame, wheelchair, is considered independent.)

1. Shopping
2. Walking around outside (around the house or to the neighbors)
3. Dressing and undressing
4. Going to the toilet

### Physical Fitness

5. What mark does the patient give himself/herself for physical fitness? (scale 0–10)

### Vision

6. Does the patient experience problems in daily life due to poor vision?

### Hearing

7. Does the patient experience problems in daily life due to being hard of hearing?

### Nourishment

8. During the last 6 months has the patient lost a lot of weight unwillingly? (3 kg in 1 month or 6 kg in 2 months)

### Morbidity

9. Does the patient take 4 or more different types of medicine?

### Cognition (perception)

10. Does the patient have any complaints about his/her memory or is the patient known to have a dementia syndrome?

### Psychosocial

11. Does the patient sometimes experience emptiness around him/her?
12. Does the patient sometimes miss people around him/her?
13. Does the patient sometimes feel abandoned?
14. Has the patient recently felt downhearted or sad?
15. Has the patient recently felt nervous or anxious?

**Sum**

Scoring:

- Questions 1–4: Independent = 0; dependent = 1
- Question 5: 0–6 = 1; 7–10 = 0
- Questions 6–9: No = 0; yes = 1
- Question 10: No and sometimes = 0; yes = 1
- Questions 11–15: No = 0; sometimes and yes = 1

## Appendix 2: Items of Short Nutritional Assessment Questionnaire Score

### Item Score

**Table:**

1. Did you lose weight unintentionally?	
More than 6 kg in the last 6 months	3
More than 3 kg in the last month	2
2. Did you experience a decreased appetite over the last month?	1
3. Did you use supplemental drinks or tube feeding over the last month?	1
